# Supplementary figures and images for: Anthracyclines induce cardiotoxicity through a shared gene expression response signature
Source: PLoS Genet. 2024 Feb 28;20(2):e1011164. doi: 10.1371/journal.pgen.1011164 (PMC10927150; doi:10.1371/journal.pgen.1011164)

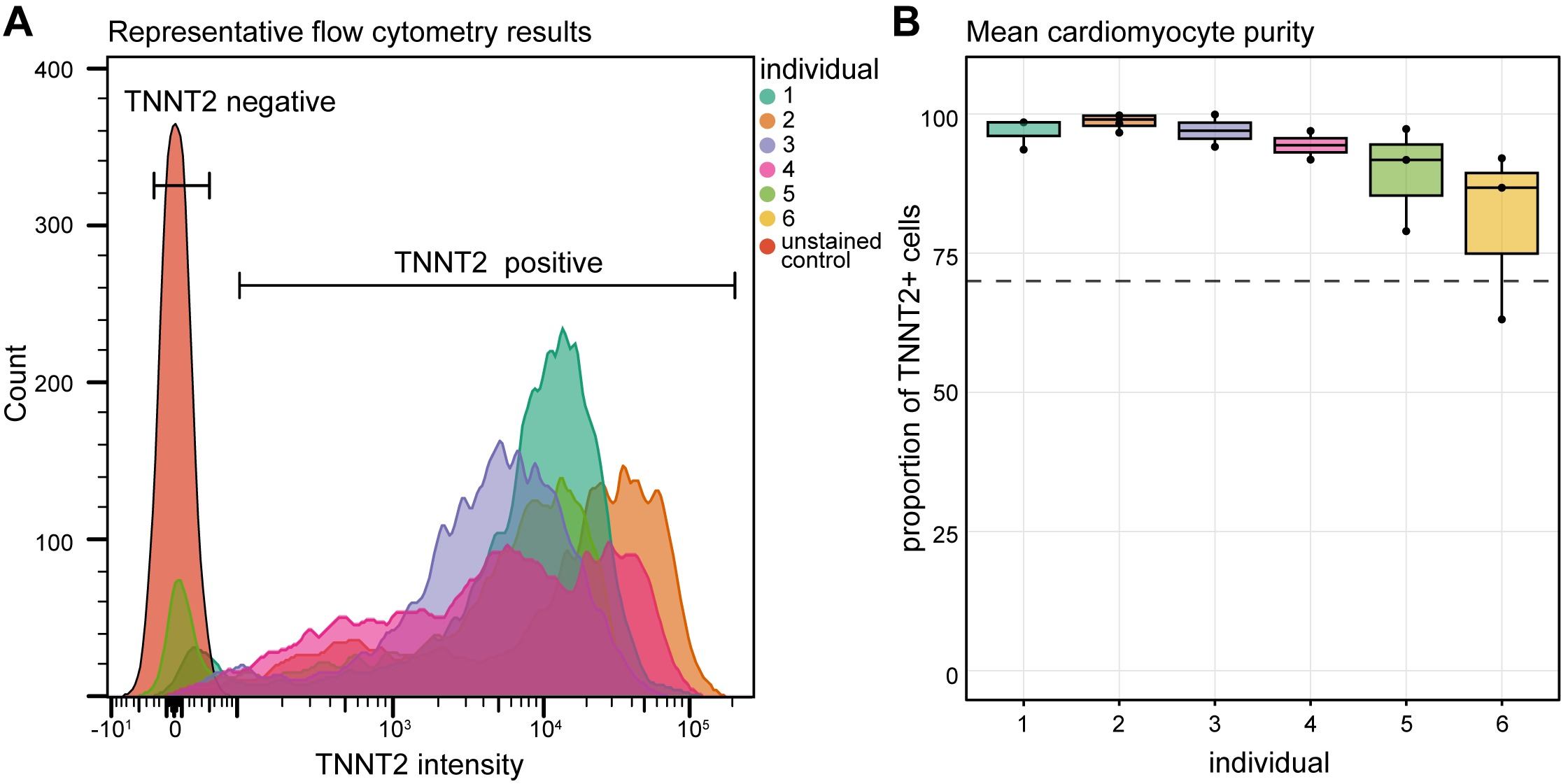

Supplement: S1 Fig — (A) Representative image of flow cytometry data indicating the proportion of TNNT2 positive cells in one differentiation experiment for each individual based on the fluorescent intensity of the phycoerythrin-labeled TNNT2 antibody and a sample of unlabeled iPSC-CMs (red cell population). (B) Percentage of cells that are positive for expression of TNNT2 for each individual. Data representative of three independent differentiation experiments used for the two drug dose-response curves, and RNA collection. The dashed line represents high-purity iPSC-CMs (> 70% TNNT2 positive). (TIF) [file pgen.1011164.s001.tif]

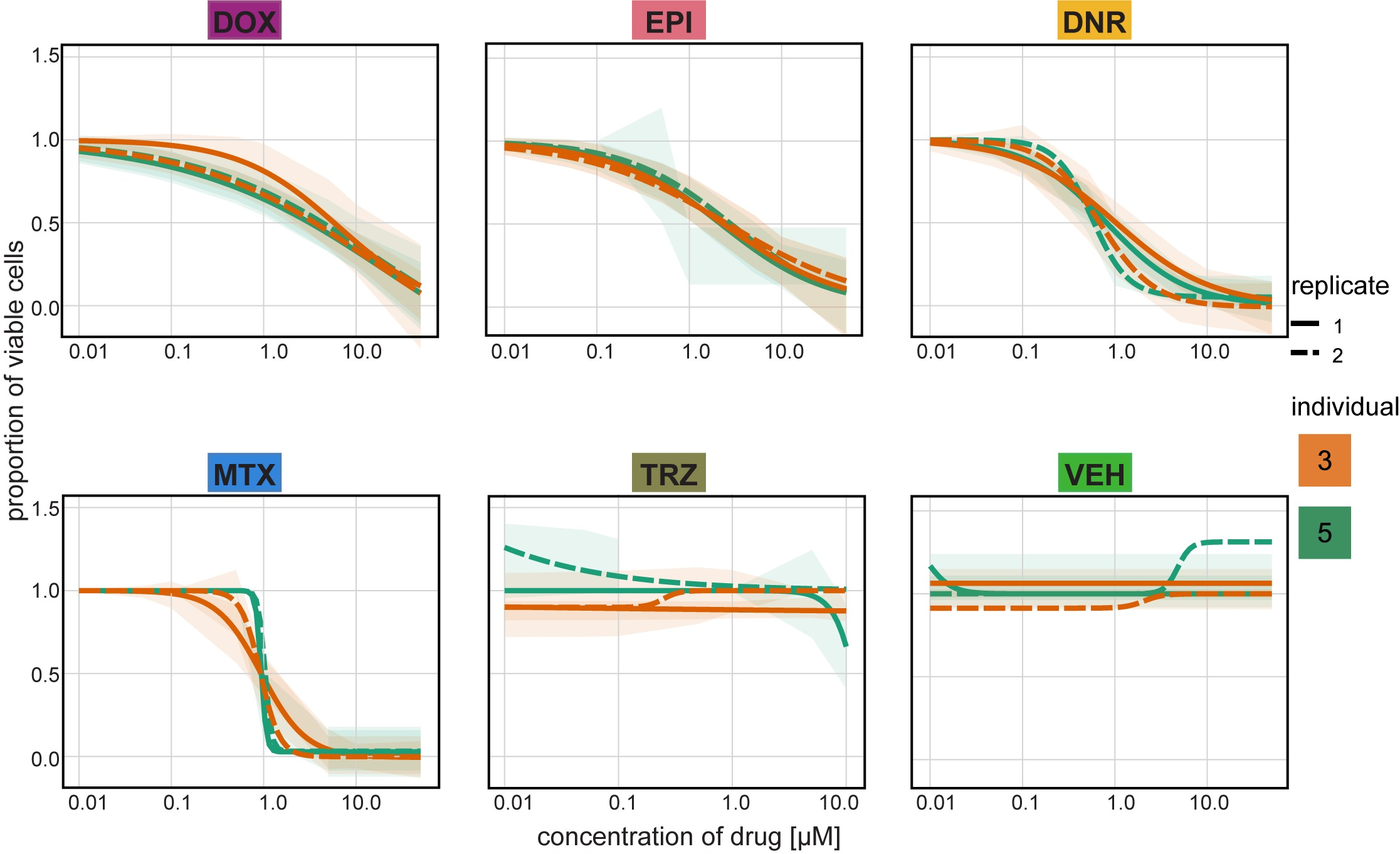

Supplement: S2 Fig — Proportion of viable cardiomyocytes following exposure to increasing concentrations of each drug. Cell viability in replicate one (solid line) and two (dashed line) in Individual three (orange), and Individual five (green) was assessed following 48 hours of drug treatment. Viability was determined at each drug concentration in quadruplicate, and the mean value was selected for generation of the dose-response curves using a four-point log-logistic regression with the upper asymptote set to one. Shading represents the 95% confidence interval from the regression analysis for Individual three (light orange) and Individual five (light green). (TIF) [file pgen.1011164.s002.tif]

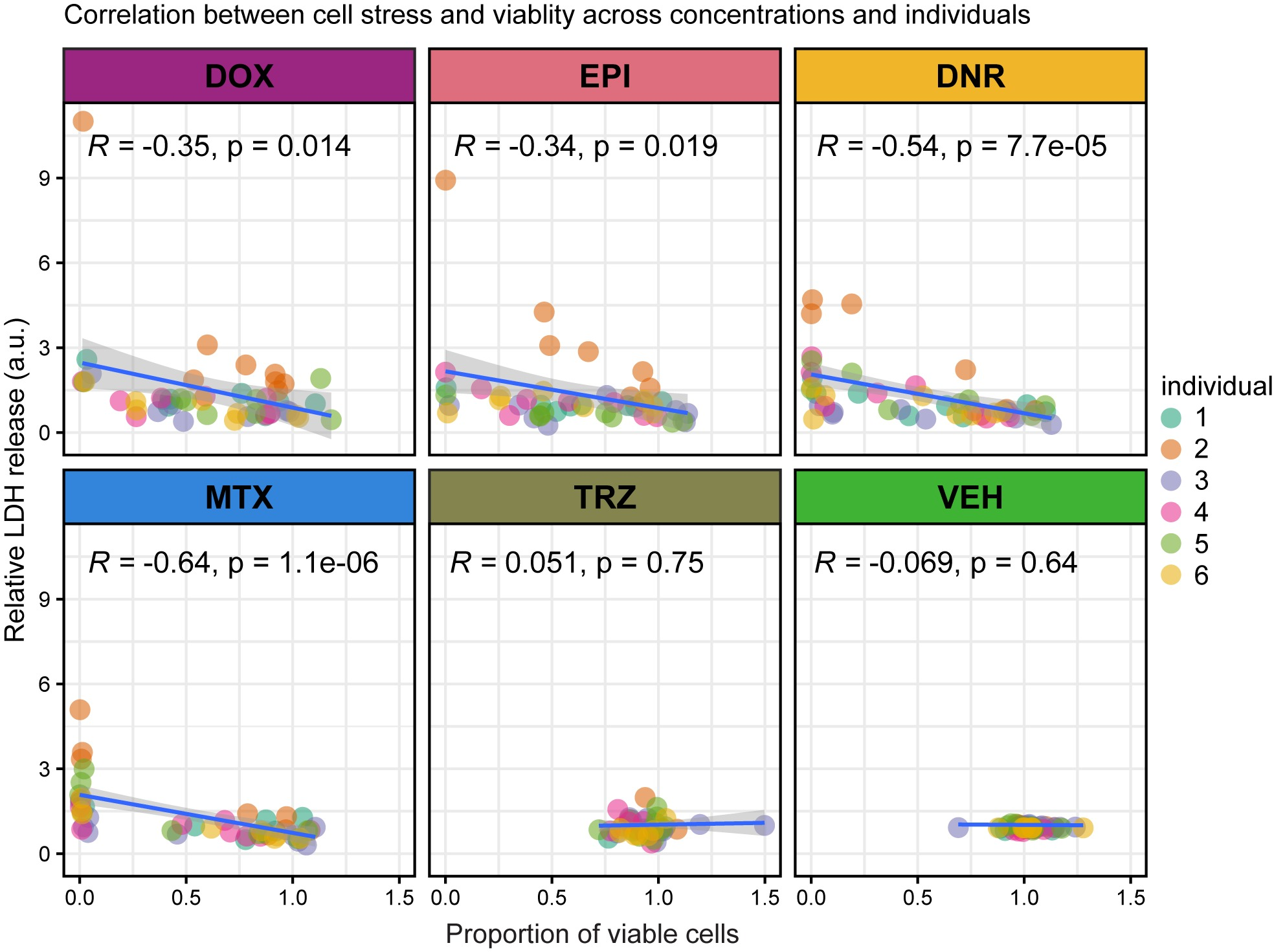

Supplement: S3 Fig — Pearson correlation between cardiomyocyte viability following drug treatment at eight different concentrations for 48 hours, and the level of lactate dehydrogenase released into the cell culture media across individuals. Data points are colored by individual (1,2,3,4,5,6). (TIF) [file pgen.1011164.s003.tif]

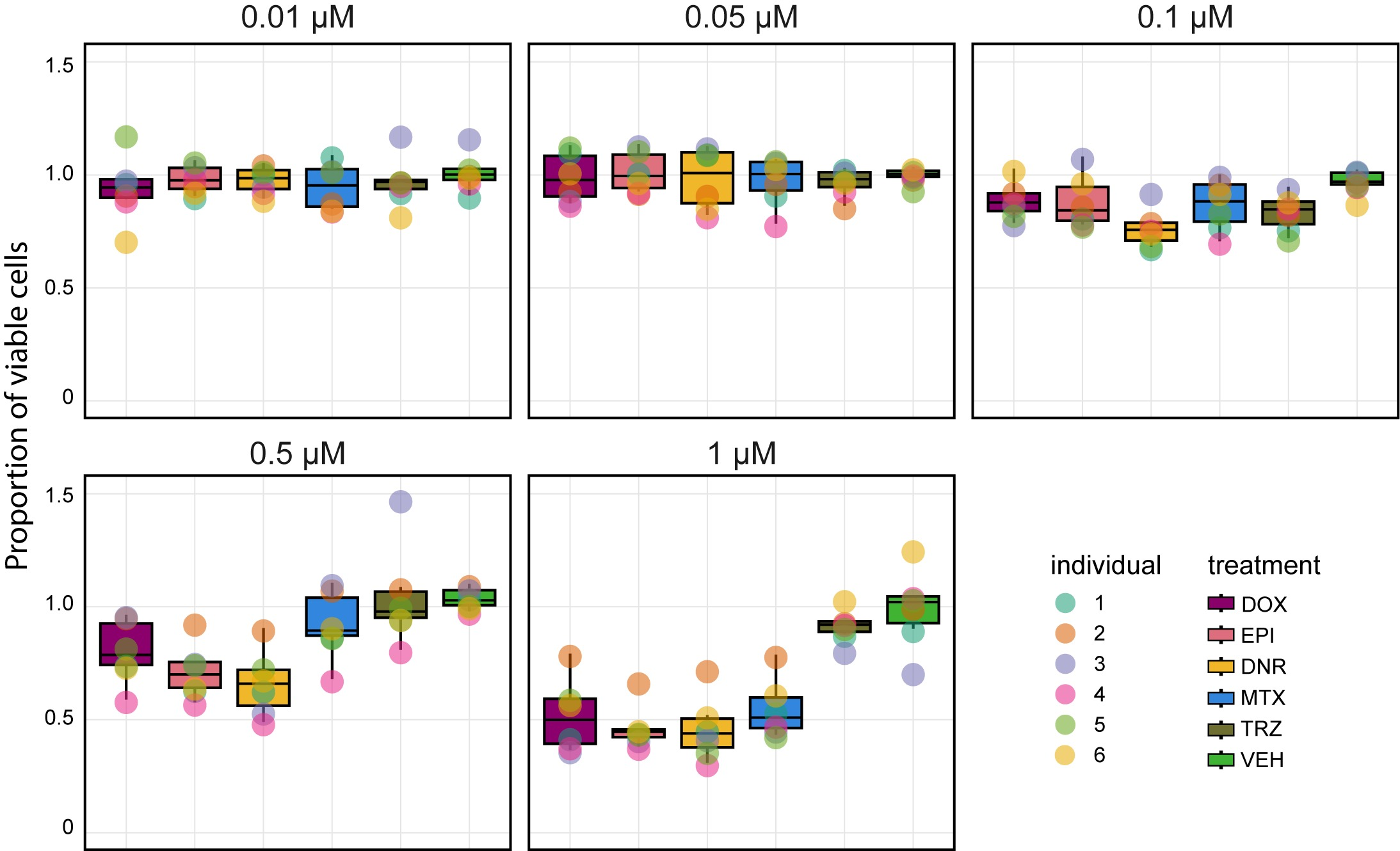

Supplement: S4 Fig — Proportion of viable cells following treatment with each drug (DOX: mauve; EPI: pink; DNR: yellow; MTX: blue; TRZ: dark green; VEH: light green) in each individual (1,2,3,4,5,6) at five sub-micromolar drug concentrations. (TIF) [file pgen.1011164.s004.tif]

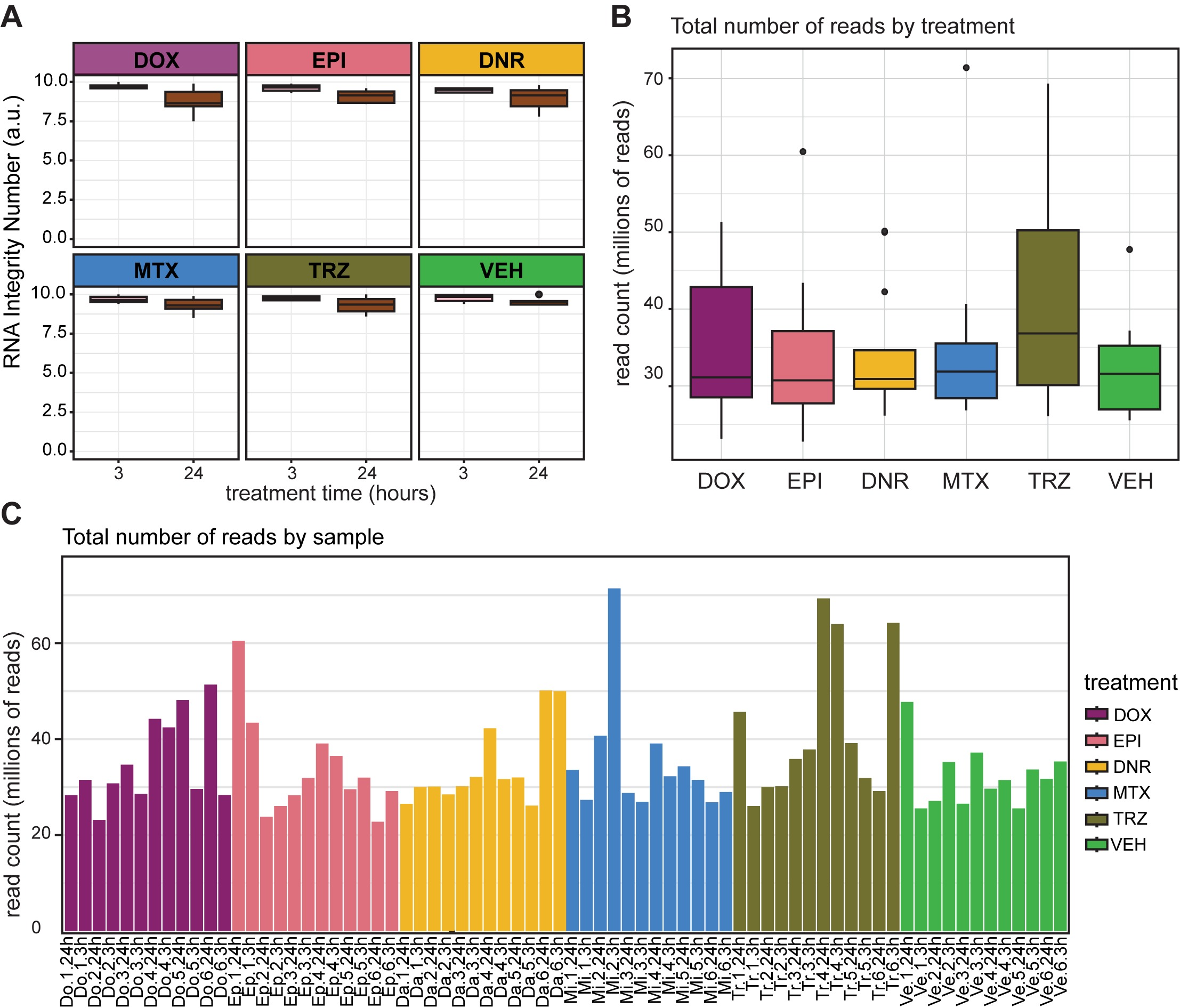

Supplement: S5 Fig — (A) RNA integrity score for each sample categorized by drug type and drug treatment time. Data inclusive of six individuals. (B) Total number of RNA-sequencing reads categorized by treatment type (DOX: mauve; EPI: pink; DNR: yellow; MTX: blue; TRZ: dark green; VEH: light green). Each drug treatment category includes data from six individuals across two time points. (C) Total number of RNA-seq reads for each of the 72 samples. Each sample is denoted by drug.individual.timepoint. (TIF) [file pgen.1011164.s005.tif]

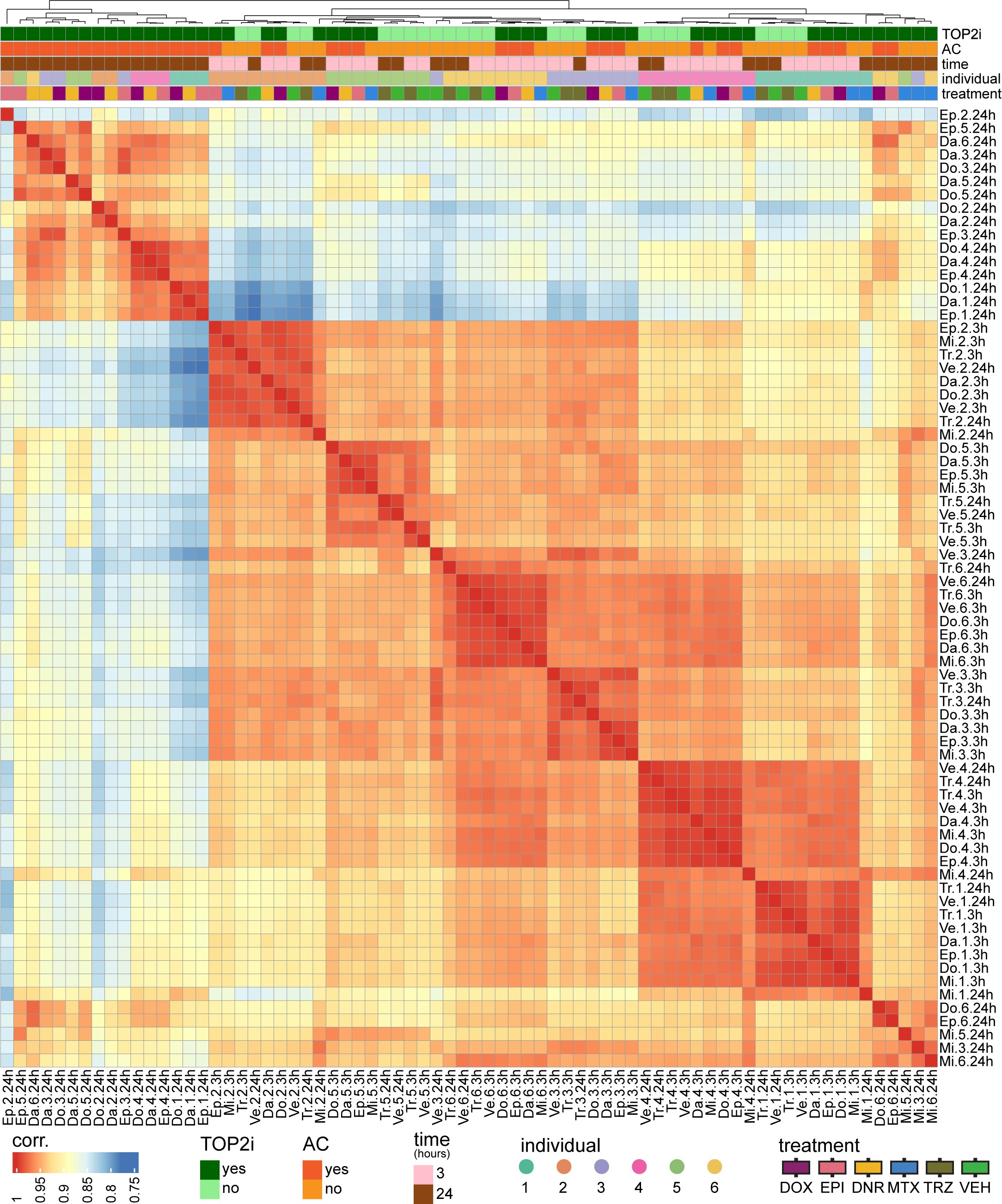

Supplement: S6 Fig — Pearson correlation of log2 cpm values across all pairs of samples. (TIF) [file pgen.1011164.s006.tif]

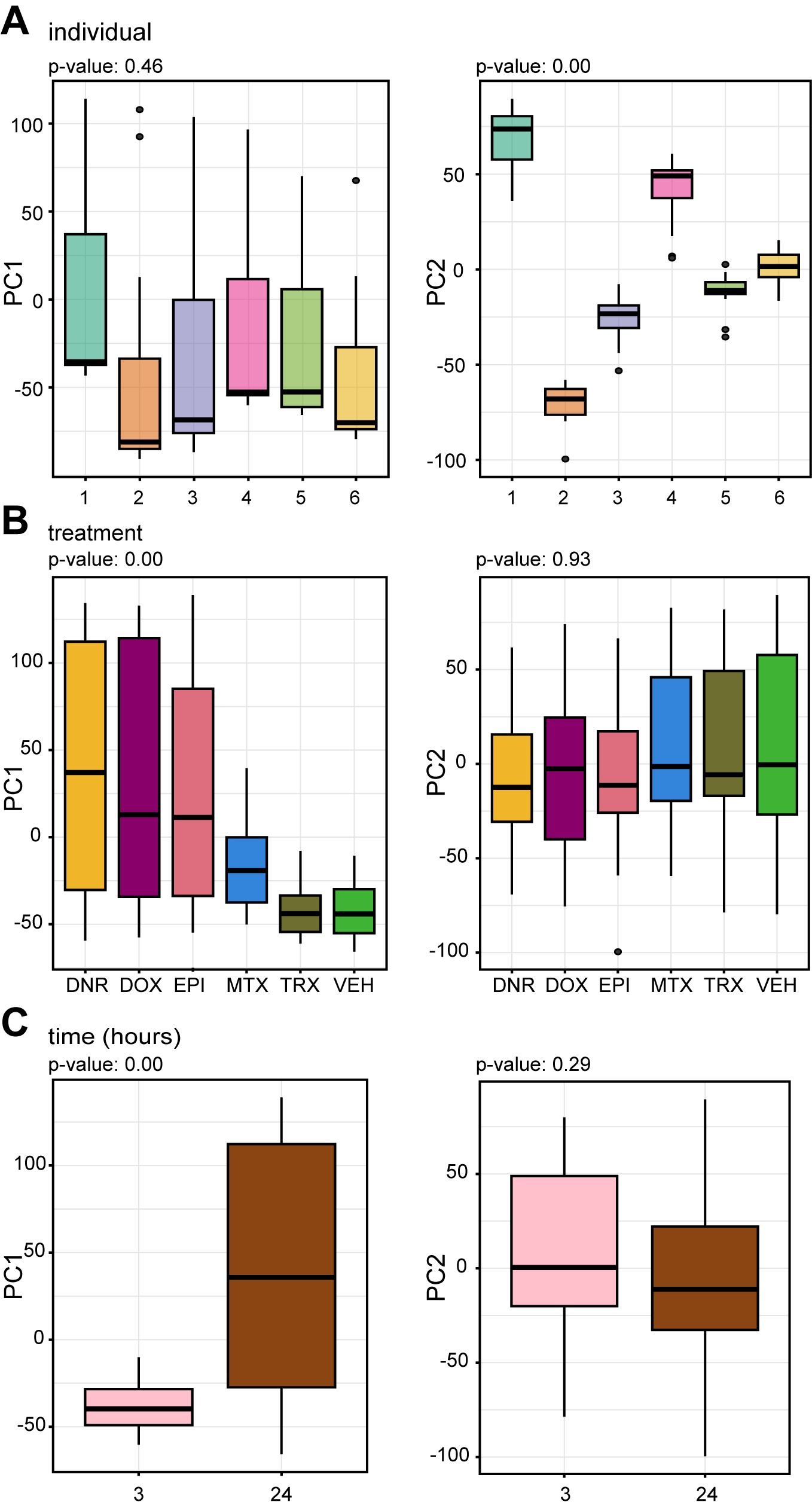

Supplement: S7 Fig — Demonstration of variance contributed to the first two principal components from three major covariates in the study: individual, treatment, and time. (A) Variance of individual as a function of PC1 and PC2. The correlation between individual and each PC is calculated using a linear model. P values represent the significance of the F-statistic from the model. (B) Variance of treatment as a function of PC1 and PC2. (C) Variance of time as a function of PC1 and PC2. (TIF) [file pgen.1011164.s007.tif]

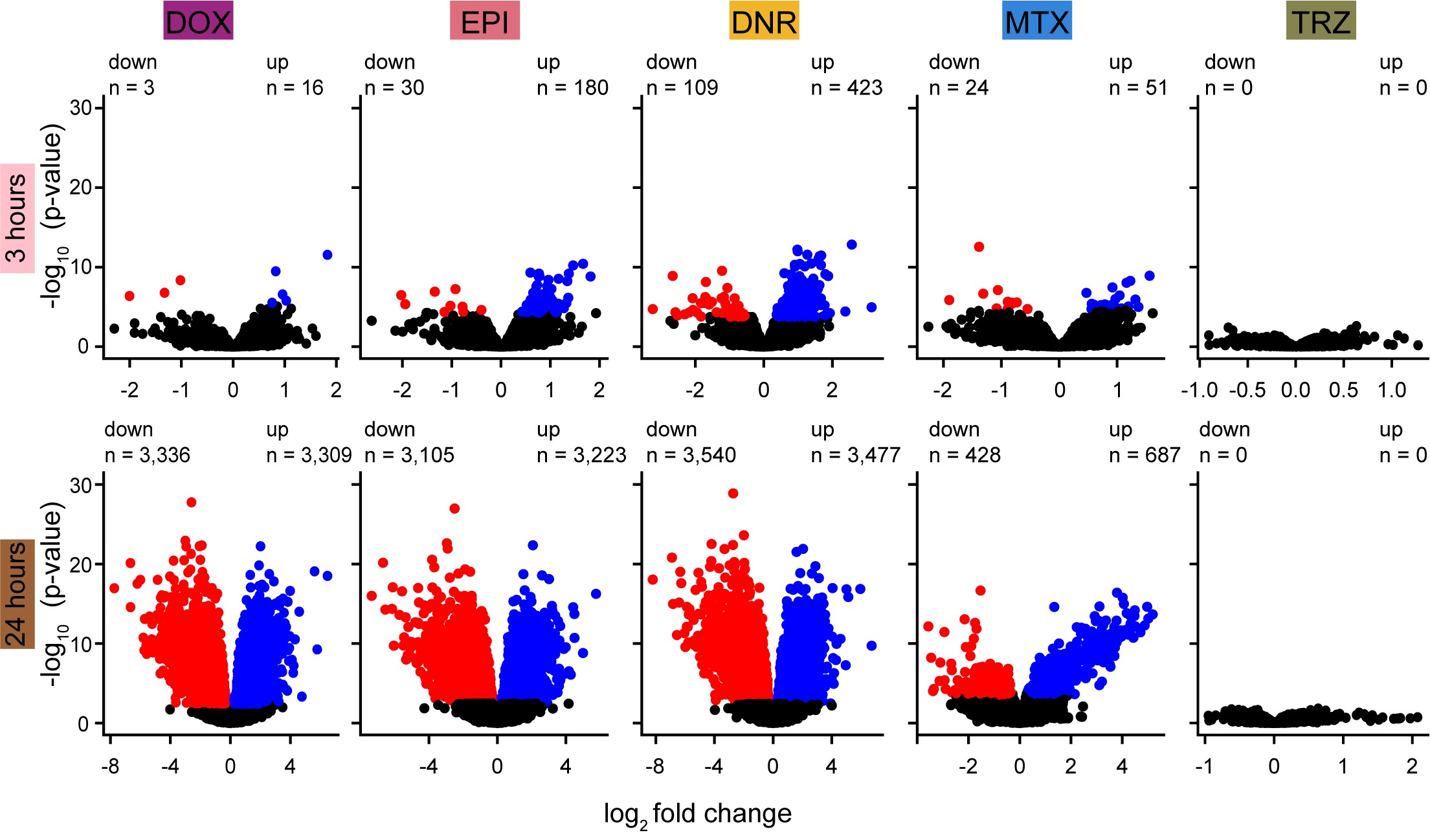

Supplement: S8 Fig — Volcano plots representing genes that are differentially expressed between drug and vehicle treatment at each timepoint. Genes that are significantly up-regulated in response to treatment (adjusted P value < 0.05) are represented in blue, and genes that are significantly down-regulated are represented in red. The number of genes that are up- and down-regulated is given for each plot. (TIF) [file pgen.1011164.s008.tif]

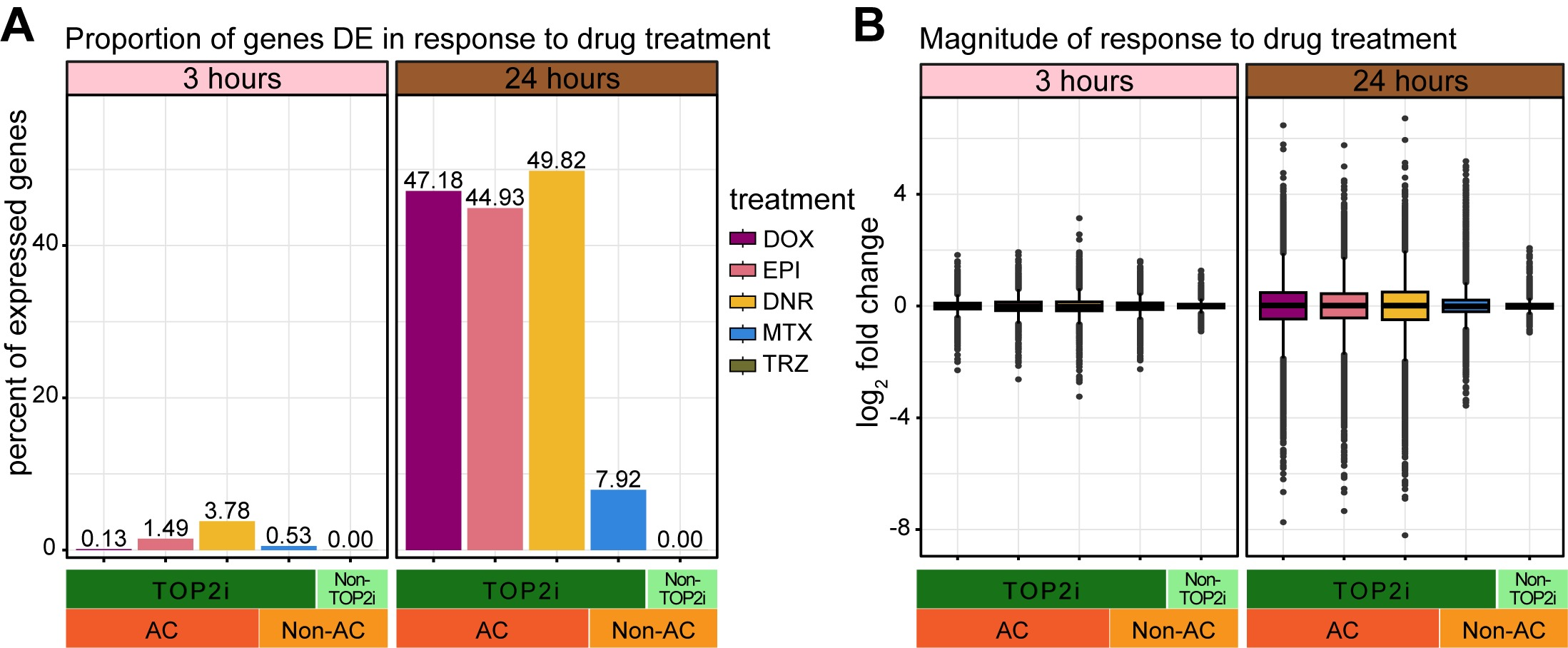

Supplement: S9 Fig — (A) Percentage of genes that are differentially expressed between each drug treatment and the vehicle following three and 24 hours of treatment. (B) Log2 fold change between drug-treated and VEH-treated samples for all 14,084 expressed genes following three and 24 hours of treatment. (TIF) [file pgen.1011164.s009.tif]

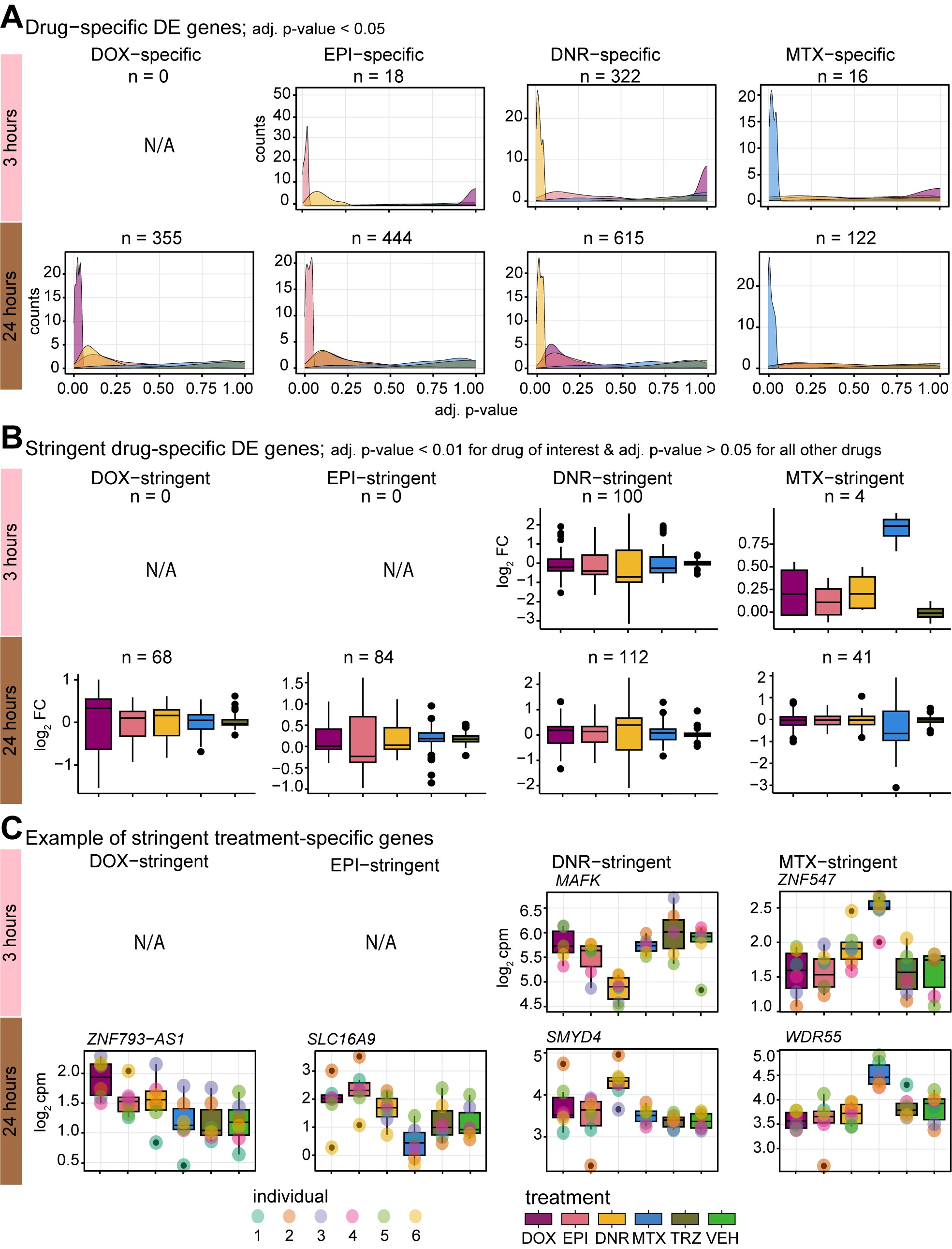

Supplement: S10 Fig — (A) Distribution of adjusted P values for all genes classified as drug-specific response genes based on overlap of significantly differentially expressed genes meeting the adjusted P value cutoff of 0.05 across drugs at three and 24 hours. P values are shown for all drug treatments for the set of drug-specific genes (DOX: mauve; EPI: pink; DNR: yellow; MTX: blue; TRZ: dark green; VEH: light green). (B) The log2 fold change of all genes meeting a stringent adjusted P value cutoff of 0.01 for the drug of interest & > 0.05 for all other drugs to identify drug-specific response genes at three and 24 hours. (C) Examples of expression levels of stringently-identified drug-specific response genes across drug treatments at each time point. (TIF) [file pgen.1011164.s010.tif]

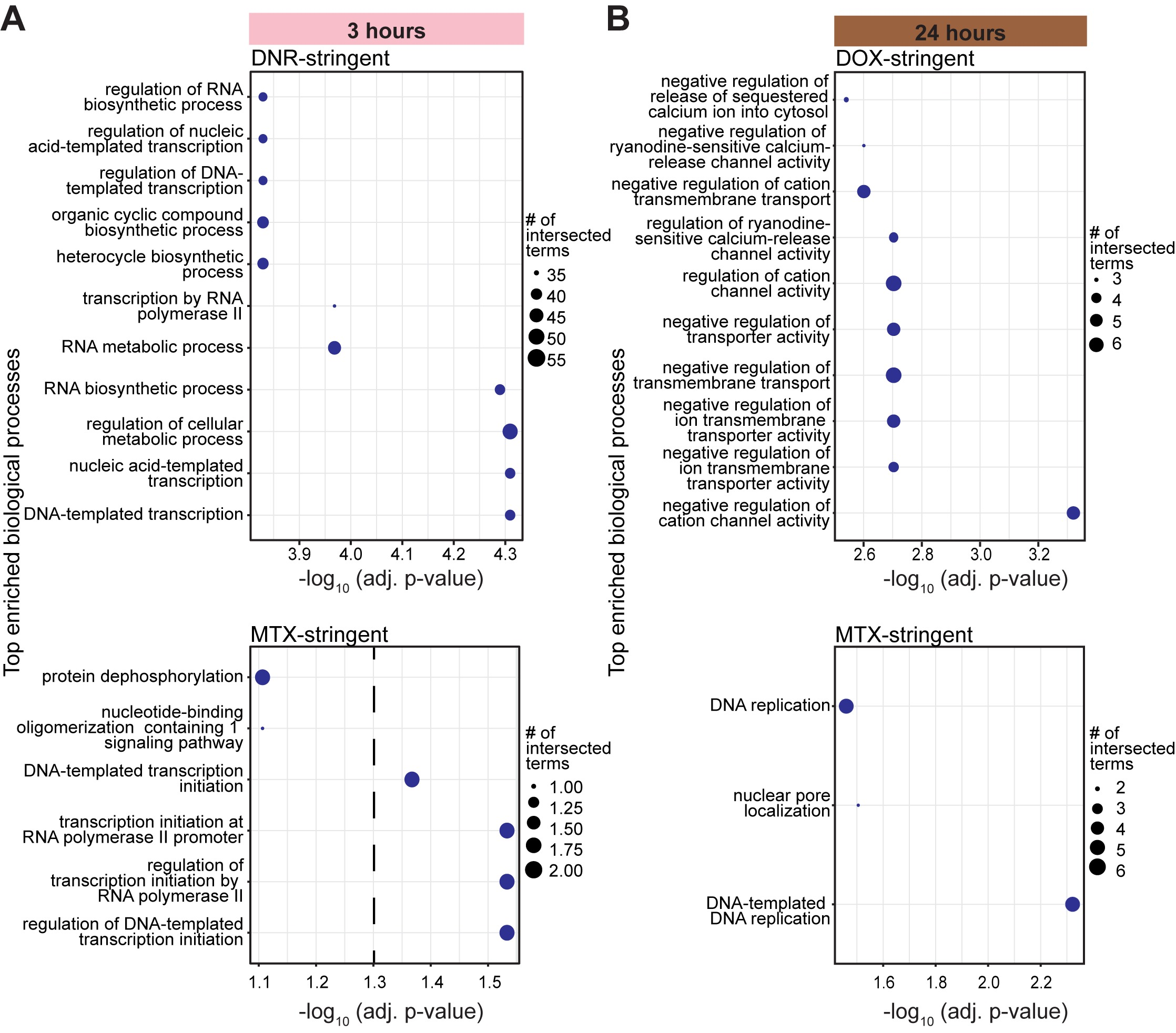

Supplement: S11 Fig — (A) Biological processes enriched amongst genes classified as stringent DNR-specific and stringent MTX-specific response genes compared to all expressed genes following three hours of treatment. The top ten most enriched biological processes from Gene Ontology analysis that meet an adjusted P value cutoff of 0.05 are shown except for MTX-stringent where only processes to the right of the dashed line are significantly enriched. Dot size represents the number of stringent drug-specific response genes that are annotated as belonging to the particular biological process. There are no DOX-specific or EPI-specific response genes that pass the stringent threshold at three hours. (B) Biological processes enriched amongst genes classified as stringent DOX-specific and stringent MTX-specific response genes compared to all expressed genes following 24 hours of treatment. There are no EPI-specific or DNR-specific response genes that pass the stringent threshold at 24 hours. (TIF) [file pgen.1011164.s011.tif]

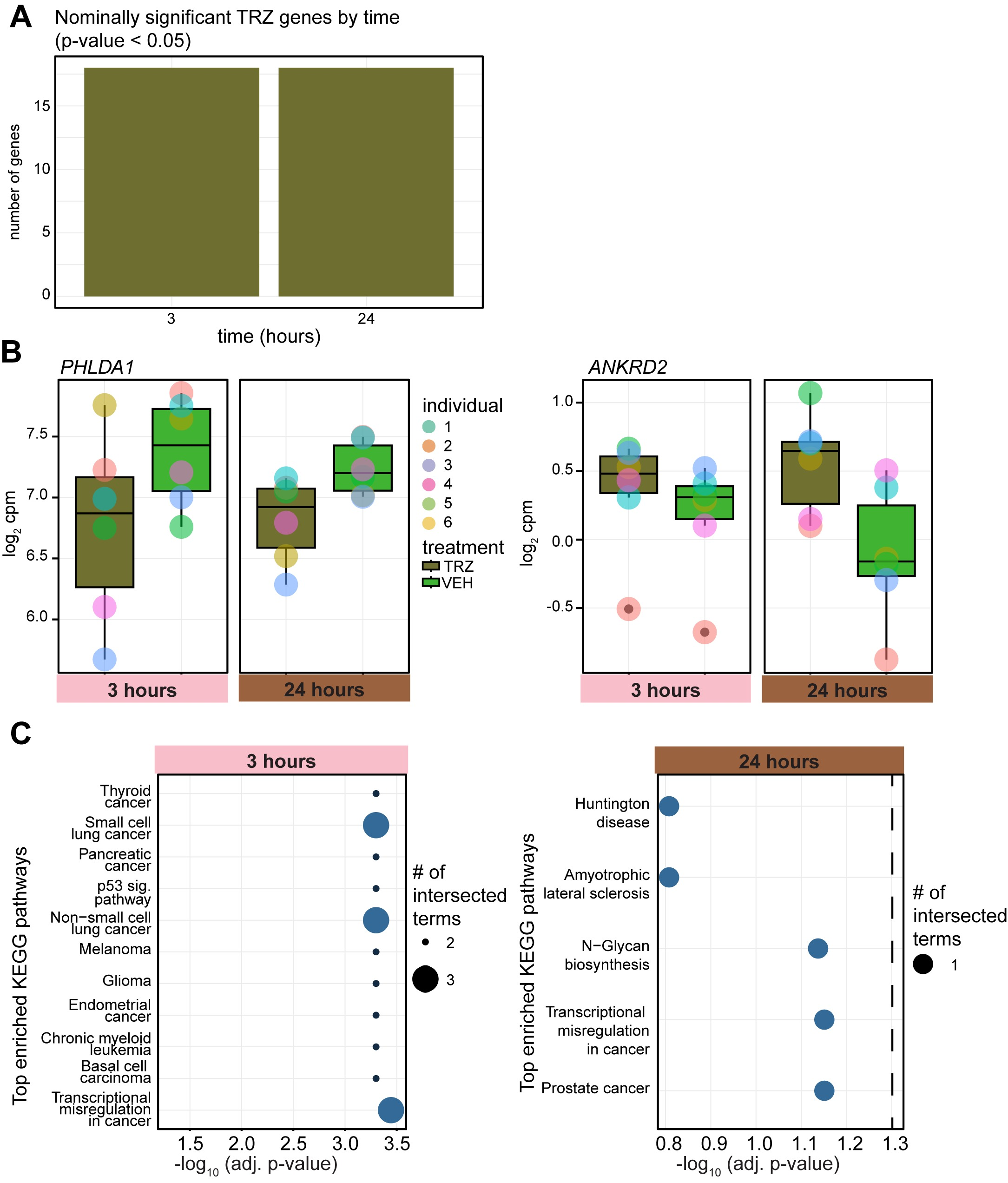

Supplement: S12 Fig — (A) Number of TRZ response genes that pass a nominal p-value cutoff (unadjusted P < 0.05) at three and 24 hours. (B) Expression of PHLDA1, a three hour TRZ response gene, and ANKRD2, a 24 hour TRZ response gene. (TRZ: dark green; VEH: light green). (C) Top KEGG pathways represented amongst TRZ response genes at three and 24 hours. Dashed black line is -log10 P < 0.05. (TIF) [file pgen.1011164.s012.tif]

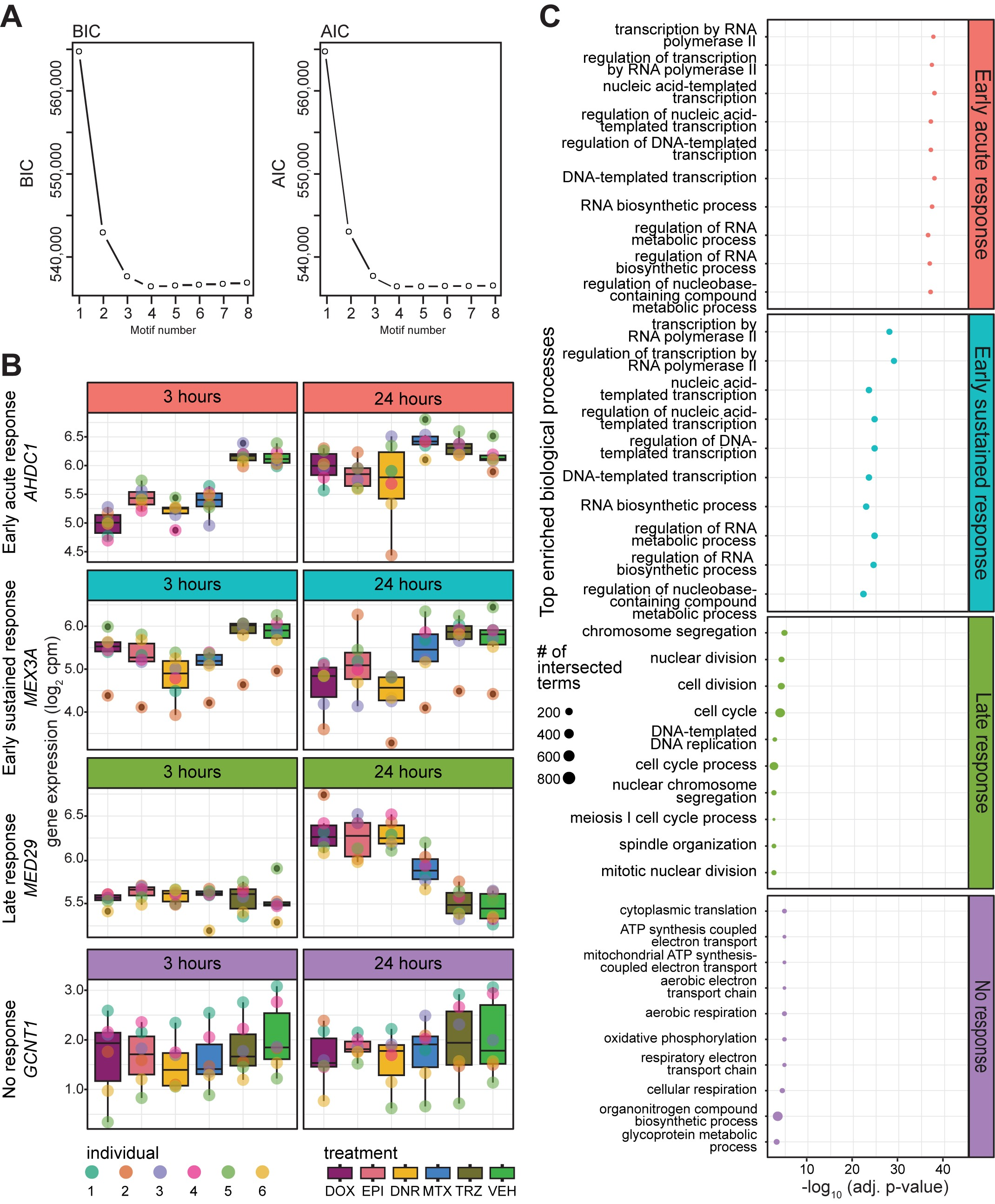

Supplement: S13 Fig — (A) Bayesian information criterion (BIC) and Akaike information criterion (AIC) at increasing numbers of Cormotif correlation motifs following joint modeling of pairs of tests. (B) Gene expression levels of genes assigned to each TOP2i response signature in each drug treatment at each time point. The AHDC1 gene represents the Early-acute response motif (red), the MEX3A gene represents the Early-sustained response motif (blue), the MED29 gene represents the Late response motif (green), and GCNT1 represents the No response motif (purple). (C) The top ten most enriched biological processes (adjusted P value < 0.05) that are enriched in the response gene categories compared to all expressed genes. Dot size represents the number of correlation motif genes that are annotated as belonging to the particular biological process. (TIF) [file pgen.1011164.s013.tif]

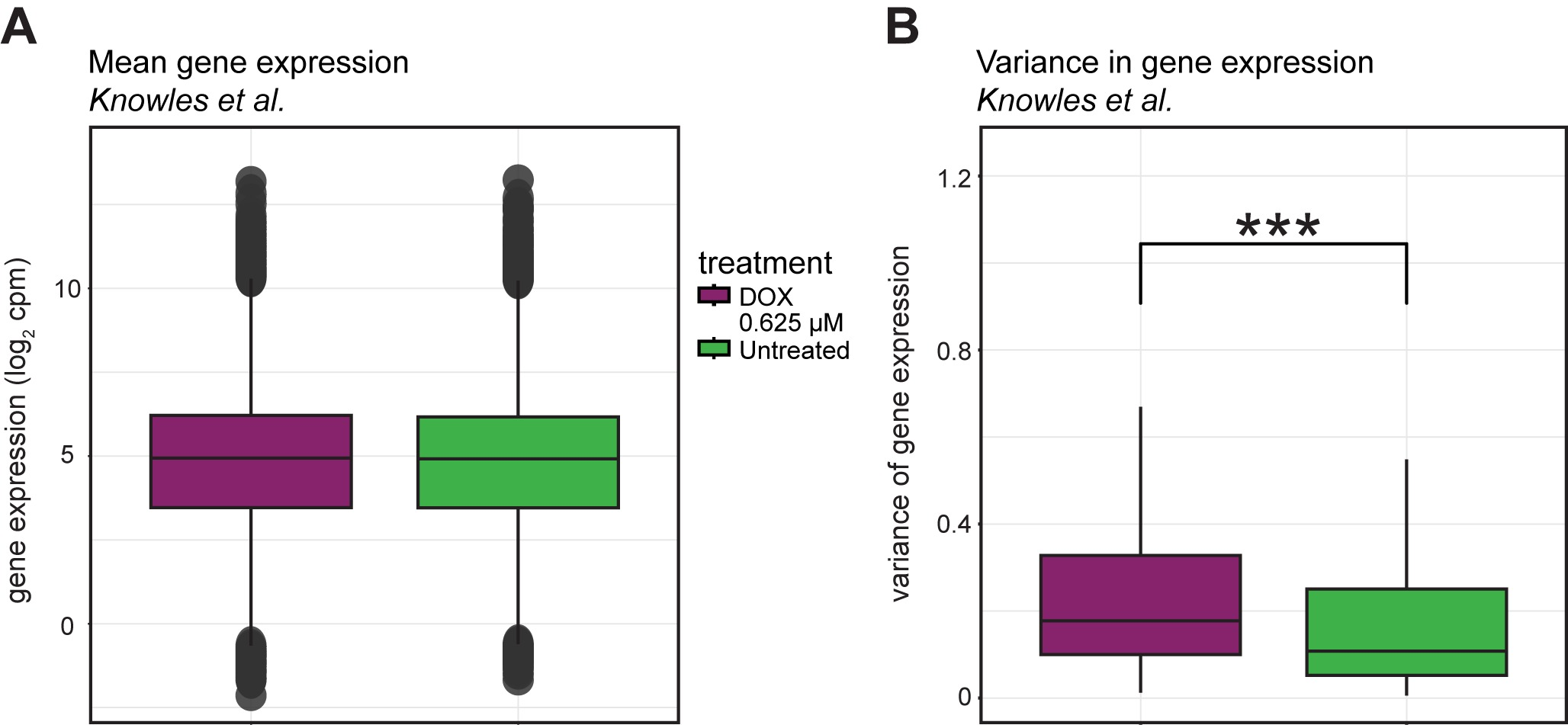

Supplement: S14 Fig — (A) Mean of 12,317 expressed genes in untreated and 0.625 μM DOX-treated samples [15]. (B) Variance of gene expression across 45 individuals in untreated and 0.625 μM DOX-treated samples. Asterisk indicates P < 0.001. (TIF) [file pgen.1011164.s014.tif]

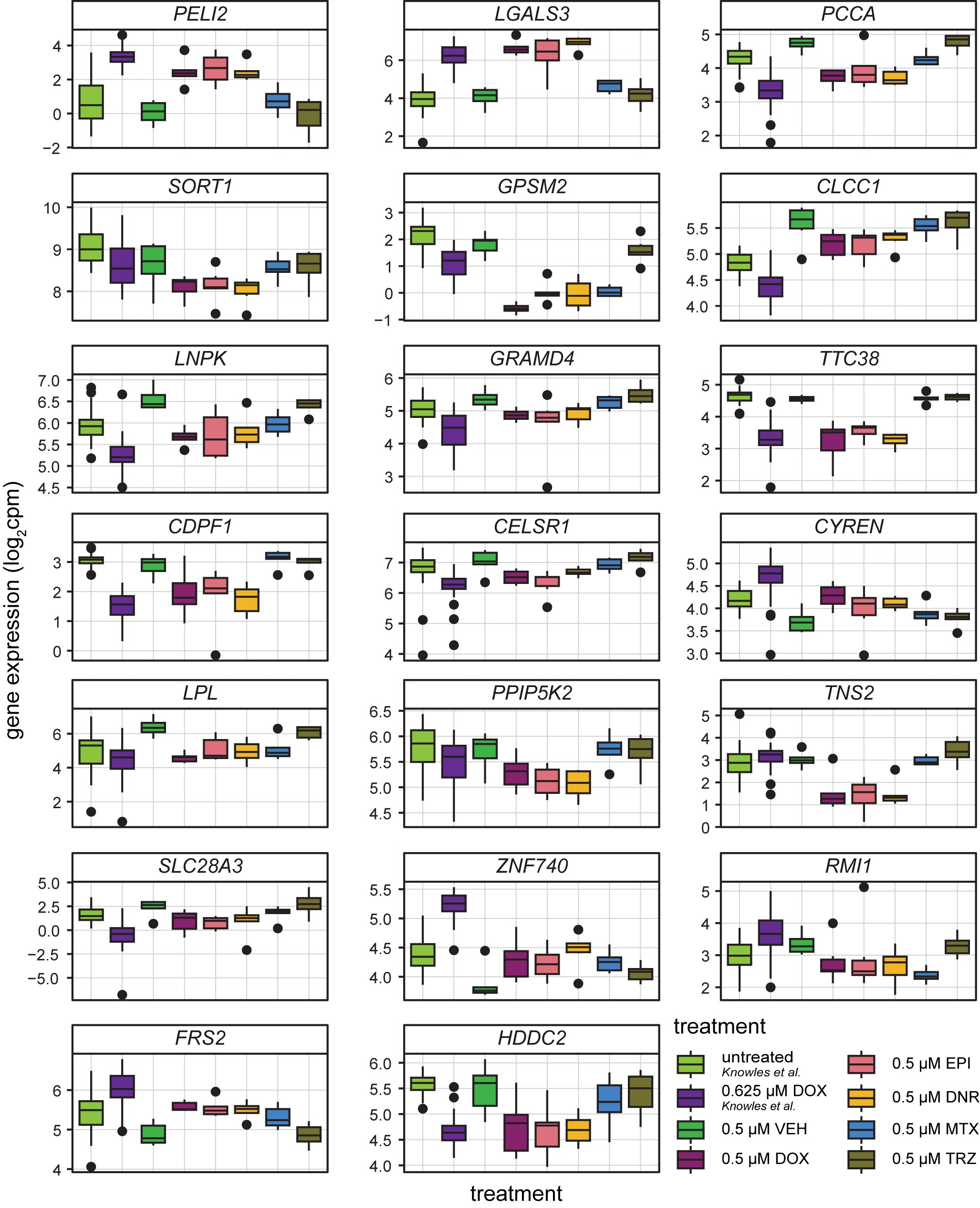

Supplement: S15 Fig — Gene expression levels of DOX-responsive genes in cardiotoxicity-associated loci in our data (VEH: light green; DOX: mauve; EPI: pink; DNR: yellow; MTX: blue; TRZ: dark green), and in DOX-treated samples across 45 individuals (untreated: lime and 0.625 μM DOX: purple) [15]. (TIF) [file pgen.1011164.s015.tif]
