# Supplementary material for: Anthracyclines induce cardiotoxicity through a shared gene expression response signature
Source: PLoS Genet. 2024 Feb 28;20(2):e1011164. doi: 10.1371/journal.pgen.1011164 (PMC10927150; doi:10.1371/journal.pgen.1011164)
Supplement: Attachment — Submitted filename: Matthews_R_Reviewer_comments.pdf [file pgen.1011164.s031.pdf]

Dear Editors

Thank you for sending the reviews. We have addressed your comments as well as the reviewers' comments as detailed below. We believe that these changes strengthen the manuscript. In particular we have (i) included a citation for the Reyes paper and discussed how our DOX results corroborate their findings, (ii) added further analysis on the TRZ data and dedicated a section in the Discussion section of the paper to discuss the results further, (iii) highlighted the novelty of our study, (iv) removed text, figures and data not key to the central message, (v) expanded on the GWAS analysis to include additional SNPs to increase the utility of the paper as a resource for the community. We believe that these changes improve the manuscript and hope that you will find our paper suitable for publication.

As per journal requirements, we have provided both a clean version of the revised manuscript as well as one with track changes. The line numbers in the responses below refer to the version with track changes.

Editors:
the revised manuscript should clearly address the following points:
1) a citation to the paper by Reyes et al 2018, as discussed by Reviewer 3, and a discussion of how the previous findings corroborate or illuminate the results reported in this manuscript, despite the obvious differences in sample size (1 vs 6), the larger number and types of drug used in this manuscript, etc.

Indeed, we are not the first to investigate global gene expression changes in response to DOX in iPSC-CMs and already cite two such papers in the manuscript. Reyes *et al*. treated iPSC-CMs from one individual with three concentrations of DOX (50, 150, and 450 nM) and collected cells for RNA-seq at three timepoints post treatment (0, 2, 7, 12 days) giving 13 total samples (Reyes *et al.*, Tox App Phar, 2018). Each sample was run on two lanes of the sequencer to generate two sequencing replicates. It is not clear from the methods how differentially expressed genes were identified in a single biological replicate per timepoint of a single individual. We were not able to find the code, or raw or processed sequencing files associated with the paper to investigate further. Nevertheless, the goal of this study was to determine dose- and time-dependent changes in gene expression in response to DOX in cardiomyocytes from a single female individual. The authors identify a set of genes that vary across doses and time and find cell cycle and DNA damage pathways to be affected.

Our study differs in several significant ways:
-our code and raw and processed sequencing data are available with the idea of making our study a useful resource for the community.
-we include six distinct individuals to robustly identify genes responding to drug treatment and gain insight into variability between individuals.
-our study compares DOX to other breast cancer drugs that have similar and differing mechanisms of action. We compare DOX to the anthracyclines DNR and EPI, MTX, a similar TOP2 inhibitor, and TRZ an unrelated monoclonal antibody.
-we were particularly interested in early gene expression changes that occur directly in response to treatment and not secondary effects and therefore investigated responses within hours rather than days.
-we measure cellular phenotypes in addition to gene expression including viability, calcium handling, stress marker release.

-we integrate our data with other datasets relevant to anthracycline-induced cardiotoxicity including GWAS and gene expression data from individuals treated with anthracyclines, and eQTLs from cardiomyocytes treated with DOX.

We do find that genes that respond to DOX in our study (500 nM dose at three and 24 hours post treatment) tend to respond to all TOP2 inhibitors tested, and these genes are enriched in pathways related to p53 signaling and the cell cycle. This corroborates the Reyes study which investigated DOX alone. It also suggests that the main pathways affected by DOX are similar across several drug concentrations (50, 150, 450, and 500 nM). We have now put our pathway result into context with the Reyes study in lines 358-360. We also now highlight the utility and novelty of our work as a resource in lines 1036-1046, in line with Reviewer 1's comment.

2) a discussion of the observed lack of transcriptional response to TRZ and its implications for the experimental system and for the overall results.

We treated six individuals with each of the TOP2i and TRZ at two timepoints. Six individuals (i.e. six biological replicates of each drug and vehicle treatment) should provide sufficient power to identify gene expression changes in response to treatment. Indeed, we identified thousands of gene expression changes in response to TOP2i. However, we did not identify gene expression changes in response to TRZ using either a pairwise linear model or jointly modeling the data across drugs.

We have now investigated the lack of TRZ response further. We first used an alternative multiple-testing correction method, which can be more sensitive to subtle effects (q-value) and again did not identify any gene expression changes. Even at a nominal p-value cutoff of 0.05 we only identify 36 genes responding in either timepoint. We do observe a significant enrichment in pathways related to transcriptional regulation and cancer in the three hour nominal response genes. We include this new analysis in the manuscript, which is represented in S12 Fig and described in lines 376-390.

While we had a paragraph discussing our TRZ results in the Discussion in the previous version of the manuscript, we have now dedicated a section to the lack of response to TRZ and discussed these results in the context of the other drug treatments in lines 836-871.

Reviewer #1

1. At first glace the title of the paper is a bit difficult to understand/digest. I would suggest rethinking it (I am not insisting on this).

We agree that the title is too long. We have simplified it to be:
'Anthracyclines induce cardiotoxicity through a shared gene expression response signature' in lines 1-2.

2. Are the induced cardiomyocytes proliferative? TOP2A in general would only be expected to be expressed during the cell cycle, thus, if these in vitro cardiomyocytes are proliferating, and cardiomyocytes in vivo are not proliferating, the general results may skew towards a misleadingly large involvement of TOP2A in cardiotoxicity. Consistent with this TOP2A expression is far higher in the in vitro cardiomyocyte system than in left ventricle heart tissue (per the authors own result, line 252; seems to be a 60 fold difference…). TOP2A may be playing no role at all in vivo (as it is

likely not expressed). This may be worth commenting upon. Perhaps the in vitro system would be more faithful if the cells were arrested (I'm not saying the authors need to do that here).

We do not believe that our cardiomyocytes are proliferating based on our observations by eye of iPSC-CMs in culture for up to 20 days. When performing the cell viability assays, we measured the viability prior to treatment and 48 hours after treatment in a subset of differentiations to ensure even plating of cells. In this short time period, across four independent differentiations, there is no difference in viability of the vehicle-treated iPSC-CMs ($p=0.428$) suggesting they are not proliferating in this time (Author Response Image 1).

Author Response Image 1

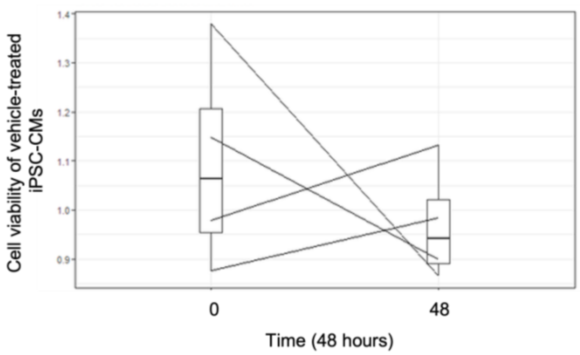

We also investigated the expression of all genes in the KEGG cell cycle annotation in our data as well as other iPSC-CM data and GTEx left ventricle heart tissue expression data where we define expressed as mean $\log_2$cpm>0. We found that 150/156 cell cycle genes are expressed in our data, 146/156 in the Knowles *et al*. iPSC-CM data, and 120/156 in GTEx left ventricle heart tissue. This suggests there may be some differences between iPSC-CMs and heart tissue.

To more easily compare the expression of *TOP2A* and *TOP2B* in our data to that in heart left ventricle (GTEx expression was based on $\log_{10}$TPM in our original manuscript), we obtained $\log_2$cpm values from 432 GTEx heart left ventricle samples.
Median expression in our data: *TOP2B*=7.85 and *TOP2A*=6.39, *TOP2B:TOP2A*=1.23
Median expression in GTEx data: *TOP2B*=6.248 and *TOP2A*=-0.8, *TOP2B:TOP2A*= 7.81

We clarified the expression of *TOP2A* and *TOP2B* in our iPSC-CMs compared to left ventricle heart tissue in the text and now discuss this as a limitation of the model in the discussion in lines 1021-1027. We have removed the analysis and figure of *TOP2A* and *TOP2B* mRNA expression from the results as it is distracting from the main message.

3. Some of the conclusions could benefit from being a bit more specific, e.g. line 419-420 "These results imply that treatment induced variability in expression across individuals in a subset of genes" comes across as somewhat weak. The paper may actually benefit from removing some of the less convincing analyses (i.e. this could easily be a 5-6 figure paper without losing much).

We have removed what we believe was the weakest data and analysis from the paper- the correlation between expression and cardiotoxicity in Figure 9 (Fig9C: TNNI+LDH release, Fig 9D: correlation between gene expression and cardiotoxicity). This was also done in response to a comment from Reviewer 2. We have replaced it with further characterization of the set of genes in loci associated with anthracycline-induced cardiotoxicity in line with this reviewer's assessment that the paper is a useful resource. We obtained the data from an adult anthracycline-induced

cardiotoxicity GWAS analysis. This study highlights ~100 SNPs associated with toxicity. We now investigate the expression of all genes in the most significant loci at both three and 24 hours to provide a comprehensive overview of potential gene mediators of toxicity. This analysis is in the new Figure 7, and is described in lines 572-597.

In order to strengthen the variance analysis, we obtained gene expression data from 0.625 μM DOX- and vehicle-treated iPSC-CMs from 45 individuals from Knowles *et al*. Using this data, we recapitulate the result that DOX treatment increases variability across individuals and include these results as S14 Fig, described in lines 488-492. We also removed the weaker analysis identifying the EPI variable genes and therefore the original S14 Fig and Tables S11-12.

We also re-organized the figures by combining figures with similar messages to streamline the paper and decrease the total number of figures from 9 to 7.

We have also edited the text to remove analyses not central to the message. We have removed Fig 3B (boxplots of *TOP2A* and *TOP2B*), S12 Fig (pairwise DE-time comparison), Fig 6B (boxplots of chromatin regulators), S15 Fig (correlation between gene expression and cardiotoxicity).

We have also made conclusions more specific in the text on lines 518-519 for example.

4. It would likely be worth highlighting the utility of the data generated as a resources for other investigators who are interested in studying AC included cardiotoxicity. This seems like a major selling point of the data that I don’t think has been emphasized.

Thank you for highlighting this strength of our study. We have added this to the discussion in lines 1036-1046.

Reviewer #2

1. Figure 9D – The correlation seems weak and heterogenous between toxicity score and the changes in expression of genes that have been shown to be correlated with cardiotoxicity in individuals from GWAS study. To verify that the gene expression changes are meaningful for the toxicity effects, it would be helpful to perform gain- or loss-of-function studies for these genes (TNS2, SLC28A3, RM1) in TOP2i-treated iPSC-CMs and measure the level of troponin/LDH release to see the direction of contribution of these genes to the disease phenotype.

We agree that the correlation is weak in many cases and there could be a number of reasons for this in a study with six individuals. We believe associating the expression change with the cardiotoxicity is challenging particularly when using toxicity measurements from a sub-lethal dose. We have therefore removed the cardiotoxicity correlation analysis from the manuscript. We did however seek to replicate the gene expression changes of these genes in cardiotoxicity-associated loci by comparing our data to that of Knowles *et al.* where they treated iPSC-CMs from 45 individuals with a range of DOX concentrations. We expanded our GWAS analysis as described in response to Reviewer 1 to include more loci, and find that of the 20 genes in cardiotoxicity-associated loci that respond to DOX in our data, 18 respond to 0.625 μM DOX with the same direction of effect in the Knowles data. We now include these results in S15 Fig, which are described in lines 594-597. While we decided to broaden our identification of potential target genes, rather than deeply characterize a subset, we note that since our original submission a paper, which characterizes the role of *RMI1* in the DNA damage response, was published. We now cite this paper in line 956, which adds further support for its role in mediating cardiotoxicity.

2. The data presented for gene expression changes and cardiotoxicity effects does not specifically describe whether there exists a positive/negative correlation within the six individual iPSC-CM lines but as an average of the change in all six individual iPSC-CMs. It would help to have more delineation of the phenotype of the six individuals and the data from their iPSC-CMs. While the iPSC lines are said to be from healthy female donors, do any of these individuals have a history of or risk factor for breast cancer? Have these individuals been treated with TOP2i clinically and develop or not cardiotoxicity? Is it known what their genotypes are for the GWAS-associated gene loci? From the current assays, are there any gene expression changes that tracks with the toxicity phenotype across these six individuals (e.g. highest change in gene expression correlates highest LDH/Troponin release within the six iPSC-CM lines sampled regardless of whether these are GWAS-associated genes?)

This is a good suggestion. We investigated the change in expression in response to treatment with the change in cardiotoxicity score per individual as suggested using the genes originally listed in Fig 9A. This gives us more genes with significant correlations in at least one drug treatment and includes *RARG* as the most striking (Response to Reviewer Fig 2). While there is significant signal in DOX, we see similar, but non-significant signal in response to EPI and DNR, which we believe makes it challenging to generate a strong conclusion. After careful thought, we ultimately decided to remove the analysis with the ELISA data as limited cardiotoxicity is induced at this drug concentration, and instead bolster the GWAS analysis and integration with our data.

Author Response Image 2

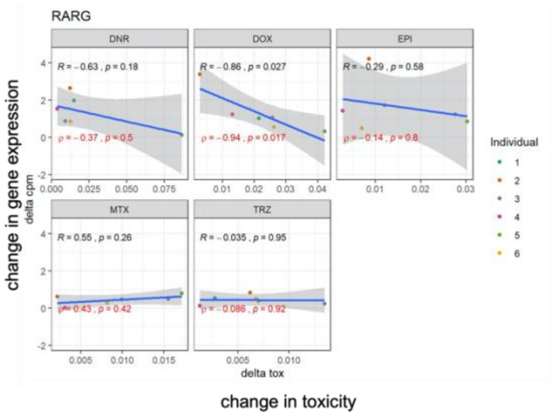

The individuals that we included are healthy females of Asian ancestry, in their 20s and 30s. The iPSCs are from a biobank and we do not know have any other personal information about them to know whether they are at risk for breast cancer, whether they have undergone TOP2i treatment etc. The idea of using young healthy individuals is that they are unlikely to have been diagnosed with breast cancer and undergone treatment. Indeed, the lack of clinical data on these individuals likely corresponds to the heterogeneity in response observed as described above.
We have made their ages as well as the fact that they have no known cardiac disease or cancer diagnoses explicit in the results in lines 166-169 and added all known information on each cell line in the methods in lines 1069-1075.

3. Given the large amount of gene expression data presented from many individual iPSC lines treated with many TOP2i drugs, the main message of this study can get somewhat loss with all the different gene changes with different drugs. It would be helpful if the authors can focus on a specific message (i.e. changes in X gene expression correlate the best with increase TOP2i

toxicity in iPSC-CMs where X can be chromatin regulating, base excision, or GWAS associate genes, etc).

Reviewer 1 had a similar comment about refining the message. As discussed in response to Reviewer 1, we have edited the text and removed figure panels not central to the message.

We have also reworked the GWAS figure to be more comprehensive of identified loci with the comment of Reviewer 1 in mind about the utility of the paper as a resource. We have also annotated every gene in these loci with the results of previous figures i.e eQTLs, chromatin regulators and response signatures to provide insight into potential mechanisms of these cardiotoxicity-associated loci. We believe Fig 7 now ties in several analysis pieces together. These results are described in lines 584-592.

Reviewer #3

Although a lot of results have been reported from this study, there is limited novel conclusion that could derived from this work. The transcriptome changes that induced by Dox have been studied in iPSC derived cardiomyocytes (10.1016/j.taap.2018.07.020).

We have described how our study differs to Reyes *et al*. in our response to the Editor above. In brief our study investigates transcriptome changes to six drugs, including DOX. Our data from DOX (in Fig 3F) recapitulates the main results on the types of pathways that are induced in response to DOX described in the paper above. We describe how our data corroborates the results from Reyes *et al.* in lines 358-360. We also emphasize the conclusions, novelty and clinical implications of our study in lines 1036-1046.

The manuscript summarized the general level of transcriptomic signatures from TOP2i but does not provide either molecular mechanism investigations or translational impacts.

Moreover, the abstract indicated that the main goal of this study is to investigate whether all TOP2i would behave the same way regarding their induction of cardiotoxicity. The presented results show some differences; however, in the absence of a true negative control (see detailed comment below), it is difficult to interpret what these observed differences mean. Therefore, for the most part, the current study failed to address the question it set out to address.

The goal of our study was to compare the effects of multiple TOP2i, focusing on ACs, on cardiomyocytes. This set includes three ACs and one non-AC. We also included TRZ as a non-TOP2i. We do not believe that the lack of response to TRZ has bearing on the response to ACs. It does show that the changes we observe in response to TOP2i are not due to non-specific drug effects when we treat the cells. We asked the question about whether different ACs would induce a unique gene expression response signature, which might give insight into drug-induced cardiotoxicity. Our results reveal that the gene expression response to ACs is remarkably similar indicating limited drug-specific effects. We believe that our study was designed appropriately to address the question posed. We have addressed the observed lack of response to TRZ as outlined in response to the Editor and below.

1. Study design issues:
a. The study of drug induced gene expression changes were conducted at 2 preselected time points and at a fixed concentration of 5 drugs. The authors then try to categorize expression changes across time points into four clusters. Pharmacology of drug response is a dynamic

phenomenon with time and concentration highly intertwined. It is difficult to justify the meaning of this kind of arbitrary classification in real world.

We were interested in understanding early gene expression changes induced by TOP2i prior to secondary effects that might contribute to the cardiotoxicity. To do so, we assessed cell viability at a range of concentrations to select a sub-lethal dose (Fig 1). We then performed a pilot RNA-seq experiment measuring gene expression after 3 hours of exposure of 0, 0.1, 0.5 and 1 μM DOX to select the precise concentration. This analysis revealed that 0.1 and 0 μM DOX were very similar to each other, and that the 0.5 μM-treated sample was the lowest concentration of DOX that did not cluster with the vehicle (Reviewer response Fig 2 below), which motivated further investigation of this dose.

Author Response Image 3

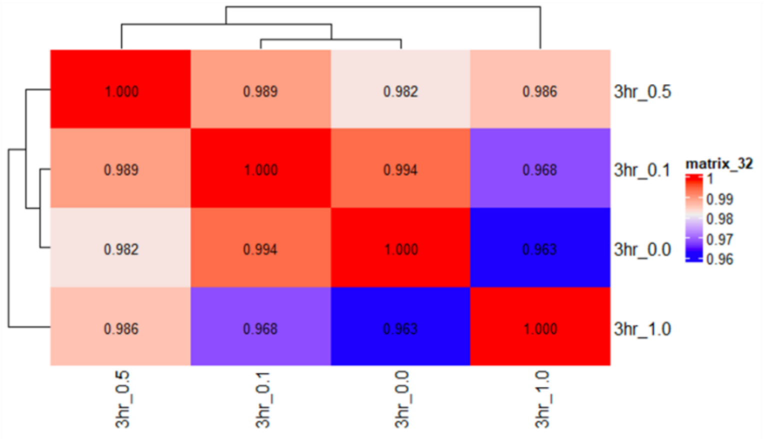

Correlation of gene expression between 0, 0.1, 0.5, 1 uM DOX-treated iPSC-CMs for 3 hours

We then performed a literature search for concentrations of all drugs in plasma of cancer patients to verify the dose that we provisionally selected (0.5 μM) fell within this range for all drugs (Table S3). We believe that because this concentration has been observed in cancer patients, it is a dose worth characterizing. At this concentration, in our time range, we transition from hundreds of differentially expressed genes at three hours to thousands of differentially expressed genes at 24 hours suggesting that this time period captures the dynamics of early responses.
Our DOX response genes are enriched in the same DOX response pathways identified by Reyes *et al*. who investigated three similar concentrations (0.05, 0.15 and 0.45 μM) suggesting robustness of results in this concentration range. We now state this in lines 358-360.
However, we do note in the discussion that there may be differences in the drug effects at different concentrations.

Our gene expression response signature analysis is performed in an unsupervised way i.e. the number of signatures that best fit the data are determined with Bayesian information criterion analysis. The result of this analysis is four. We did not *a priori* choose a pre-defined set of response signatures. We have made this clear in the results in lines 297-299.

2. Technical issues:
a. Trastuzumab (TRZ) is tested in this study as a drug that do not target TOP2. However, TRZ is also known to lead to cardiotoxicity. It is not clear why the investigators did not observe any cell death after exposing iPSC-CM cells to this drug. It is even more bizarre to see no gene expression change after TRZ treatment when compared to vehicle control. Is that because wrong dose was used, solubility issue…? The lack of expression impact is especially worrisome. The authors need to add plausible explanation to the discussion, so readers won't question the validity of the model/work. Furthermore, because this out of class drug is not working in the experimental system, the authors literally just compare phenotypic differences among TOP2is. When compare among items, there will always be differences observed. The authors presented these differences, but was not able to draw meaningful conclusions with the data.

It is true that TRZ has been shown to induce cardiotoxicity in patients. However, this damage appears to be reversible unlike AC-induced cardiotoxicity, which suggests that it may not have effects on cell viability unlike the TOP2i.

There are two papers in the literature where iPSC-CMs are treated with TRZ and their effects studied. One treats iPSC-CMs at 0.5 μM for 7 days and shows no effects on viability but that there are effects on metabolic genes by RNA-seq and effects on contraction (Kitani, Circ, 2019). The other uses 100 ug/ml (0.687 μM) TRZ and treats iPSC-CMs for 48 hours (Necela, Clin Tran Med, 2017). They do not identify any genes that pass an adjusted p-value threshold. They find 517 nominally significant genes ($p < 0.05$), which they show to be involved in metabolism. This is the context in which our study was performed.

We do not observe effects on viability after 24 hours of treatment in line with Kitani *et al*. We do not observe any significant effects on calcium, although there is a trend toward decreased amplitude and increasing rise slope in line with Kitani *et al*. (they show decreased efflux).

We do not observe any gene expression changes using an adjusted p-value threshold or q-value threshold. We have now investigated how many genes pass a nominal p-value threshold of 0.05. Using this approach, we identify 18 genes that respond at three hours and 18 genes that respond at 24 hours. We find that this set includes two of the four genes that Necela *et al*. identified at a nominal p-value threshold and validated by RT-qPCR that show the same direction of effect (*SLC6A6*, and *PHLDA1*). We note that our cell culture conditions are different as our media does not contain glucose, which could influence the differences in results. However, our results are in line with both clinical and experimental observations.

We have added the nominal p-value analysis to the paper as S12 Fig described in lines 376-390, and have expanded on the TRZ section in the discussion in lines 836-871. We also make it clear in the introduction that TRZ-induced cardiotoxicity is reversible in lines 146-148.

We have made edits to the text to make the novelty and utility of our study clear.

Additional comments:
1. The variations are large across 6 individuals. Repeated measurement of gene expression could be helpful in validating the results.

We collected pilot RNA-seq data from Individual 4 treated with 0.5 μM DOX for 3 hours. We have now correlated the expression of this pilot sample with the 0.5 μM 24 hour DOX samples from Individuals 1-6 used in the manuscript, and find that the pilot sample from Individual 4 is most

similar to the Individual 4 sample used in the manuscript (0.926 correlation) compared to all other individuals (0.871-0.921 correlation range). This result suggests that there is a strong individual component. Indeed, in the PCA analysis, 'Individual' associates with PC2 (15% of variance) clustering samples from different treatments together by individual again suggesting a strong individual component to expression.

We include six unrelated, healthy female individuals in our study that are of at least two different Asian ethnicities and between the ages of 21 and 32. We have added all the available meta data associated with each individual to better contextualize the inter-individual differences in lines 1069-1075.
We have also used a DOX-treated iPSC-CM dataset (Knowles *et al*., eLife, 2018) to compare our results to. As discussed in response to Reviewer 1, we show the increase in variance following DOX treatment is recapitulated in this data set (S14 Fig). We also investigate the expression of all DOX-responsive genes in the AC-induced cardiotoxicity loci analysis in this dataset and show that 18 of 20 DOX-response genes have a response in the same direction as our data (now in S15 Fig).

2. The transcriptomic changes upon Dox intervention have been studied in iPSC cardiomyocyte. The author has generated new data from other TOP2i but did not further investigate the findings. It would be interesting and critical to know what drug specific transcriptomic changes are.

One of the main findings in the paper is that TOP2i (DOX and DNR, EPI and MTX) induce similar gene expression changes in iPSC-CMs. Using our joint modeling approach we thought we may see signatures of drug-specific effects i.e. a set of genes changing expression in response to only one or a couple of drugs. However, this is not what our data showed. We observed a single TOP2i response signature that differed only in the timing when these changes occurred. Identifying drug-specific responses using a pairwise approach is often not an effective way to identify condition-specific effects given likely incomplete power in at least one condition. However, we used a stringent approach to identify genes that respond only in a single drug by using a two-step cutoff approach (adj $p < 0.05$ in the drug of interest and adj $p > 0.1$ in all other drugs). This analysis did reveal hundreds of drug specific response genes; however there is still some signal in several of the other drug conditions indicative of incomplete power. We show these results in S10 Fig as well as the pathways that are enriched in S11 Fig.
We characterize the gene expression changes in response to these different drugs in Figures 4-7.
We have added text on lines 324-325 to make the section on drug-specific responses more apparent and text to lines 411-412 to make the joint analysis result more clear.

3. The impact of findings from this manuscript is not clear. It is critical to know whether any of these genes have clinical impacts. The author could elaborate on this in the discussion.

We believe that our study has potential clinical applications. There is controversy in the literature about the relative effects of different ACs on the heart. In patient cohorts, individuals can only be treated with one type of AC and thus effects of different ACs on the heart are tested across different individuals. Our *in vitro* system allowed us to investigate how the same individuals respond to different treatments in a controlled environment. Our results show that, in general, genes that respond to one AC tend to respond to other ACs suggesting that individuals who experience cardiotoxicity following treatment with one type of AC are also likely to experience cardiotoxicity following treatment with other ACs.

The genes in cardiotoxicity-associated loci that respond to drugs could have an effect in the clinic. For example, one of the genes, galectin-3, has been suggested to be a potential circulating biomarker of various diseases including heart disease.
We have added text in lines 1036-1046 of the discussion about the overall potential clinical impact, as well as discussion of an example target gene in lines 956-977.

4. Do any gene candidates from DE could affect the drug effectiveness on iPSC-cardiomyocytes?

We are unsure what is meant by this comment and therefore are unsure how to respond.

5. Why only 3 individual's iPSC were selected for Calcium investigation? Especially, these three (individual 2, 3, 5) have distinct LD50 in Dox group in Figure 1C.

The calcium experiments are time-sensitive and time-consuming experiments to perform and require a specialist microscope that is not available in our lab. We therefore investigated calcium handling for three randomly selected individuals.
We now state in the manuscript that these individuals were randomly selected in lines 221-224.

6. Figure 4A, it is not clear that TRZ treatment fall into a cluster. Especially the correlation between TRZ 3h and TRZ 24h is relatively low (0.16).

This analysis relies on unsupervised hierarchical clustering that groups samples together based on their similarity to other samples (in this case correlation of log fold change between treatment and vehicle) regardless of the label assigned to the samples. We did not assign samples to clusters. It is true that the correlation between these samples is low but the other samples are sufficiently similar to each other that they are grouped together and the TRZ samples are grouped separately.
We now denote that the samples are grouped by hierarchical clustering in the main text in lines 297-299 in addition to the method being stated in the corresponding figure legend.

7. What is the potential mechanism behind the fact that HSV showing up in Figure 4D?

We mention in the manuscript that the HSV-1 pathway consists of many genes associated with p53-associated DNA damage. Specifically, the HSV-1 annotated pathway contains many C2H2-type zinc binding domain proteins that are associated with the DNA-damage response and transcriptional regulation (Vilas, Trends in Genetics, 2018).
We have added this second reference connecting genes in the HSV-1 pathway to p53-associated damage in lines 360-364.

8. For Figure 7A and 7B, could you use PCA plot (or similar plot) to show the similarities/dissimilarities of different treatment groups? It is not clear by just showing the variation in gene expression.

A PCA plot of this data is presented in Fig 3A. We are looking at a different property of the data in this figure i.e. the variance in expression across individuals in response to treatment.
We have added clarifying schematic panels to this figure (now Fig 5) to aid in interpretation.

9. lines 406-408, referring to fig 7C, the manuscript stated "…at 24 hours it clustered based on whether the drugs are ACs or not". However, it looks like all five drugs cluster together in Fig 7C. Please also show the vehicle result in this plot as well.

Similar to comment #6, this analysis is performed by hierarchical clustering. Within the 24 hour timepoint there are two distinct branches which separate the anthracyclines (DOX, DNR, EPI) from MTX and TRZ.
This analysis compares the variance in each treatment with the variance in the vehicle to generate the F statistic. This is the value that is compared across drugs.
We have added details to the heading and a schematic of now Fig 5C to aid in the understanding of the figure. We appreciate that there are several analysis steps that were not immediately clear when looking at the original figure.

10. No statistical tests on Figure 3B, 6B, 8D, and 9D.

Based on some of the earlier comments, we have removed Figures 3B, 6B and 9D from the manuscript.
We have added an asterisk to denote that *JPH3* is differentially expressed (i.e. passes an adjusted p-value threshold of 0.05) in Fig 6D (Fig 8D previously) and explained this in the legend.

11. It is very hard to understand and interpret Figure 4A. What exactly does the correlation done on? Gene expression (1 gene or all genes) or drug response (like LD50).

We have adjusted the figure (now Fig 3A) heading so it is clear it's the log fold change in expression being correlated.

12. The text is opposite to what Figure 9D shows.

Thank you for pointing this out. We did by mistake have the key upside down. Based on some of the other comments we have removed this figure panel.
